# Werner's Syndrome: Understanding the Phenotype of Premature Aging—First Case Described in Colombia

**DOI:** 10.1155/2019/8538325

**Published:** 2019-02-12

**Authors:** A. Rincón, L. Mora, F. Suarez-Obando, J. A. Rojas

**Affiliations:** ^1^Human Genetics Institute, School of Medicine, Pontifical Xavierian University, Bogotá, Colombia; ^2^San Ignacio University Hospital, Bogotá, Colombia

## Abstract

Werner's syndrome (WS) is an autosomal recessive genetic disease, which is mainly characterized by scleroderma-like skin changes, juvenile cataracts, short stature, and signs of premature aging. We report a case of a 48-year-old male patient, who presents with cardinal signs of WS including high-pitched voice, sclerotic skin lesions mainly on feet, premature greying of scalp hair, bilateral cataracts, and “bird-like” facial appearance. In addition, the patient presents other clinical characteristics observed in patients with WS such as short stature, type 2 diabetes mellitus, hypogonadism, parental consanguinity, and a history of a sibling with similar clinical characteristics.* WRN *gene sequencing identified the homozygous pathogenic variant NM_00553.4: c.2581C>T (NP_000544.2: pGln861Ter). This is the first case of WS reported in the Colombian population. We report this case to avoid misdiagnosis of this infrequent condition and allow timely identification of potential complications associated with premature aging, especially malignancies, cardiovascular and metabolic diseases.

## 1. Introduction

WS was initially described by Otto Werner in 1904. He reported 4 cases of brothers and sisters with juvenile cataracts, skin changes similar to scleroderma in the extremities, joint deformities, short stature, senile appearance, juvenile grey hair, and genital hypoplasia [1]. Since the first description, additional case reports of WS have been described worldwide, the majority being from Japan [2, 3]. The prevalence in the Japanese population is 1/20,000 to 1/40,000 [4]. The frequency of carriers of heterozygous mutations is estimated to be highest in Japan and Sardinia, being 1/166 [4] and 1/120 [5], respectively. The prevalence in Colombian population is unknown.

*WRN *gene (also called RECQL2 or REQ3) at chr 8p12 is the only known gene responsible of WS.* WRN *gene has 34 coding exons that encodes for a nuclear protein of 1,432 amino acids; this protein is a member of the RecQ DNA helicases. Multiple biochemical and cell biological studies have been made to evaluate the cellular effects associated with the loss of WRN protein function. These studies have demonstrated the importance of the helical activity of WRN protein to maintain genomic stability, including DNA repair, replication, transcription, and telomere maintenance [6].

The cells of patients with WS have been studied extensively, and some abnormalities have been identified including inability to repair DNA with double-strand breaks, abnormal telomerase dynamics, cells experiencing slow growth, and shorted life cycle [7]. Another findings that have been reported are chromosome instability, prolongation of the S phase of the cell cycle, and abnormalities in the initiation of DNA replication [8].

The loss of WRN protein function causes genomic instability, resulting in the accumulation of somatic mutations, aberrant maintenance of telomeres which may lead to cellular dysfunction, loss of proliferative homeostasis, or increased cellular loss in various tissues or cell lines. These cellular changes are probably responsible for the clinical characteristics of early aging and tumor development observed in WS patients [6, 9].

Different types of mutations have been reported since the first description of* WRN *gene in 1996 [10]. Homozygous or compound heterozygous loss of function mutations in the* WRN *gene causes classical WS. Currently, there are 83 pathogenic variants reported from all over the world in the International Registry of Werner Syndrome (Seattle, WA) and the Japanese Werner Consortium (Chiba, Japan) [11]. Ethnicity-specific* WRN *mutations have also been reported in certain populations including Japanese (c.3139-1G>C, r.3139 3233del95; c.1105C>T, p.R369*∗*; c.3446delA, p.E1149fs), Sardinian (c.2089-3024A>G, r.2088 2089ins106), Indian/Pakistani (c.561A>G, r.557-654del98), Moroccan (c.2179dupT, p.C727fs), Turkish (c.3460-2A>G, r.3460 3572del113), and Dutch (c.3590delA, pN1197fs) populations [12, 13].

The majority of the pathogenic variants result in WRN protein truncation, due to exon skipping associated with stop codons, small insertions/deletions, or splicing mutations. Most of pathogenic variants are in exons, but intronic variants have also been reported [12]. These mutations produce loss of the nuclear localization signal at the C-terminal of the WRN protein and/or promote nonsense-mediated mutant mRNA decay [11].

Although a report of a possible genotype–phenotype correlation of follicular carcinoma with C-terminal* WRN *mutations and papillary carcinoma with* WRN-*N-terminal mutations among Japanese WS patients has been published [14], in general the clinical phenotypes and natural history of WS patients appear to be very similar among* WRN *mutation types and different ethnic groups [11].

The revised diagnostic criteria for WS [3] include the following cardinal signs: progeroid changes of hair, cataracts, changes of skin, intractable skin ulcers, soft-tissue calcification, bird-like facial appearance, and abnormal voice. These patients may have other associated signs and symptoms such as abnormal glucose and/or lipid metabolism, deformation and abnormalities of the bone, malignant tumors, parental consanguinity, premature atherosclerosis, hypogonadism, short stature, and low bodyweight. Genetic analysis of the* WRN *gene is now included in the diagnostic criteria.

In our patient, the diagnosis of WS was made based on the revised diagnostic criteria for WS [3]. The patient had all cardinal signs and symptoms (scarce and gray hair, bilateral cataract, changes of skin, skin ulcers difficult to manage, calcification of the Achilles tendon, bird-like facial appearance, and high pitched voice). Also, short stature, flat feet, truncal obesity, type 2 diabetes mellitus, hypertriglyceridemia, hypogonadism, and parental consanguinity were found. Confirmation of the clinical diagnosis was made by analysis of the* WRN *gene which revealed a pathogenic homozygous variant NM_00553.4: c.2581C>T (NP_000544.2: pGln861Ter). This mutation generates a stop codon at position 861 and has been classified as pathogenic.

## 2. Case Report

We present the case of a 48-year-old male, who was evaluated by the medical genetics service because he had noticed weakening of his voice with a high pitch since age 35, associated with premature graying since his 30s and skin lesions since about the age of 40. At the age of 32, bilateral cataracts were diagnosed and at 44 he was diagnosed with diabetes mellitus, currently on oral hypoglycemic agents. Additionally, he has hypothyroidism and hypertriglyceridemia in management and calcification of the Achilles tendon. Patient endorses lack of an early adolescent growth spurt; however, final stature is similar to his other 3 siblings (164 cm). Patient reports he had no child by choice.

Patient is product of the union of consanguineous parents (second cousins) and has a 49-year-old brother with similar clinical characteristics, including voice changes since the age of 28, bilateral cataracts at age 29 (subsequently presents complications from corneal ulceration and is currently legally blind), and premature graying since age 33, moreover, scleroderma-like skin changes since his 30s and diagnosis of type 2 diabetes mellitus at age 35. His brother also endorses no child by choice. No other complications such as atherosclerosis, dyslipidemia, hypertension, osteoporosis, or tumors were reported.

Unfortunately, patient's brother and parents declined genetic testing. There are no other relatives with clinical suspicion of WS.

Patient states maternal aunt has unspecified type leukemia and father with a history of acute myocardial infarction at age 65 and a diagnosis of melanoma at age 85. Maternal uncle diagnosed with lung cancer at age 72 and maternal grandfather with prostate cancer diagnosed at age 73.

**Figure 1:**
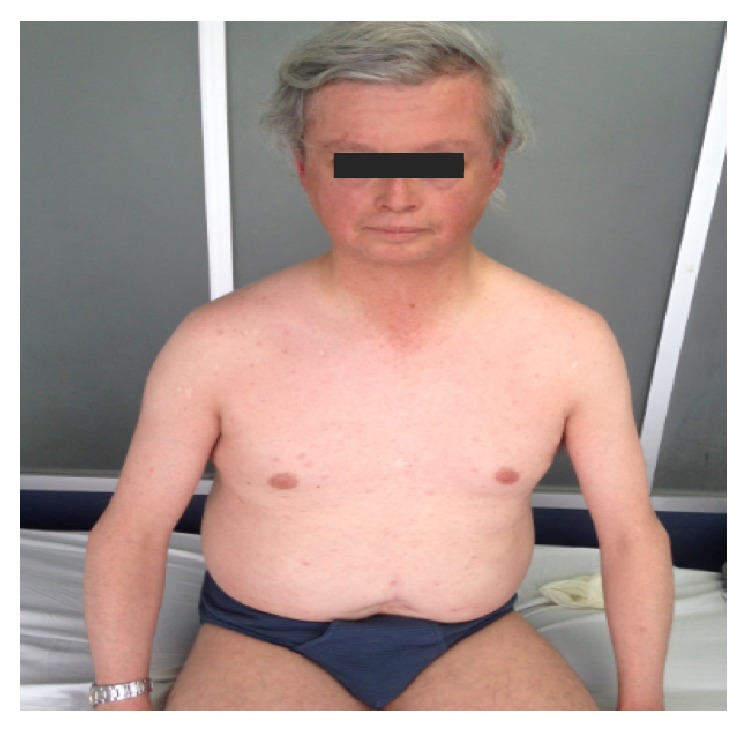
Patient's face shows “bird-like facial appearance”, beak-shaped nose, scarce and gray hair, and eyebrows. Note thin upper limbs with decreased subcutaneous fat and truncal obesity.

**Figure 2:**
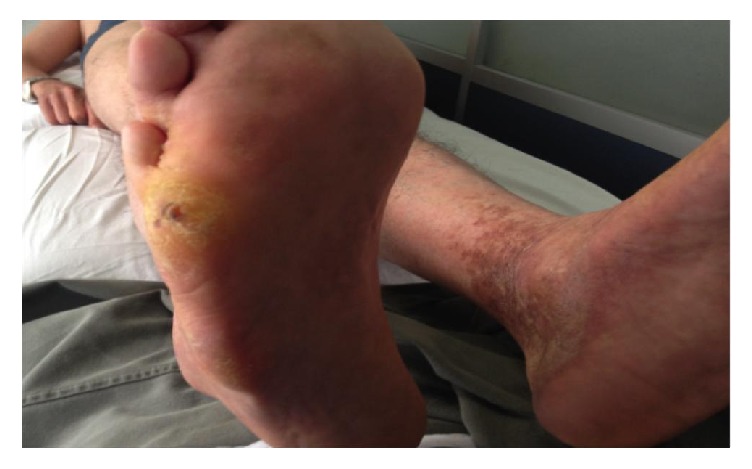
Lower limbs with markedly atrophied skin and subcutaneous fat, abnormal pigmentation of the skin and hyperkeratosis in the left perimalleolar area, flat feet, plantar hyperkeratosis, and callus in the right 5th metatarsal head and 5th metatarsal base areas.

**Figure 3:**
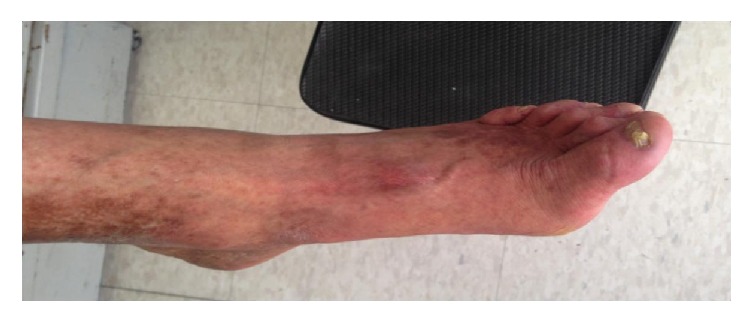
Left foot showing contractures, nail dystrophy, dermal atrophy, circumscribed hyperkeratosis and hyperpigmentation, and decreased subcutaneous fat and muscle.

On initial physical examination, he appeared much older than his age with “bird-like” facial appearance, beak-shaped nose, and bilateral cataracts, his voice was high-pitched and his hair and eyebrows were scarce and markedly gray. He had thin upper limbs with decreased subcutaneous fat and truncal obesity (Figure 1). Moreover, we found short stature, hypogenitalism, lower limbs with markedly atrophied skin and subcutaneous fat, abnormal pigmentation of the skin and hyperkeratosis, and flat feet (Figures 2 and 3).

*WRN *gene sequencing identified the homozygous variant NM_00553.4: c.2581C>T (NP_000544.2: pGln861Ter).* WRN *gene sequencing report can be found in Supplementary Material S1. This variant generates a stop codon at position 861 and has been classified as pathogenic and previously described in homozygous status in a Caucasian patient from the United States in 2006 [15].

### 2.1. Investigations

Laboratory findings included normal renal function, high blood glucose (164 mg/dl), elevated glycosylated hemoglobin (9.4%), and elevated triglycerides (324.6 mg/dl) with normal cholesterol (162.4 mg/dl). EKG showed an elevation of the J point by early repolarization. Abdominopelvic CT-scan showed bilateral renal cysts, small umbilical hernia, and no fatty liver. Testicular ultrasound showed decreased bilateral testicular volume mainly left side.

### 2.2. Outcome and Follow-Up

Regular screening for malignancies is recommended for patients with WS, due to the high risk of early-onset neoplasms. Also, it is very important to rule out cardiovascular and metabolic diseases during the follow-up of these patients. Our patient is still under periodic clinical observation and follow-up. Currently, he is on treatment with oral hypoglycemic agents for DM2 with adequate glucose control and in treatment of hypertriglyceridemia. Until now no signs of atherosclerosis or cardiovascular disease have been detected. However, he was recently diagnosed with refractory cytopenia with multilineage dysplasia, a form of myelodysplastic syndrome, which has required multiple transfusions.

According to a clinical history, the patient's brother is being monitored for inadequate control of diabetes mellitus and severe skin lesions that have been difficult to treat, but no cancer has been documented.

## 3. Discussion

The first clinical sign of WS, often recognized retrospectively, is a lack of the expected pubertal growth spurt leading to relatively short stature on adulthood. However, sometimes this clinical sign is overlooked and it is usually during early adulthood (36.7_± 10.1 years) [2] when the diagnosis is made, due to other classic features. Patients with WS are normal at birth and have adequate growth and development during childhood. Thereafter, patients begin to progressively develop the typical features of WS such as an aged appearance that includes a bird-like face, gray hair, alopecia, skin atrophy and loss of subcutaneous fat and areas of hypo- and hyperpigmentation.

Complications typically beginning in the 30s, such as bilateral cataracts, arteriosclerotic diseases (cerebral hemorrhage, cerebral infarction, myocardial infarction, and arteriosclerosis obliterans), hypertension, diabetes mellitus, dyslipidemia, osteoporosis, deep skin ulcer around the ankles, calcification of the Achilles tendon, malignancies, and early loss of infertility associated with gonadal atrophy. The main causes of death at a median age of 54 years are myocardial infarction secondary to atherosclerosis, diabetes mellitus, and malignant tumors [6].

WS patients have a much higher incidence of neoplasms and the mean age at first diagnosis of neoplasms is 43.3 years ± 9.9 years (range 20–69) as evidenced by the systematic review of the literature made by Lauper et al. [16]. The main features of cancer in WS are early age of onset, high frequency of unusual types, especially sarcomas, and multiple neoplasms [17]; however common types of cancer have also been described.

Lauper's study [16] analyzed 189 WS patients with 248 neoplasms to characterize the spectrum of neoplasia in WS; 139 (74%) of these were Japan-resident patients. They found that multiple neoplasms were observed in 22% of patients with WS and the most frequent neoplasms were thyroid neoplasms (16.1%), followed by malignant melanoma (13.3%), meningioma (10.9%), soft tissue sarcomas (10.1%), leukemia and associated hematologic disorders (9.3%), and osteosarcoma (7.7%). Cancer risk was significantly elevated in Japan-resident WS patients for the six most frequent neoplasms except leukemia and the elevated risk of these neoplasms ranges from 8.9 for thyroid neoplasms to 53-fold higher for melanomas than population controls.

We would like to contribute to the literature with our clinical observation of a classic WS case; this patient was diagnosed relatively late because this syndrome was not initially suspected, perhaps because of poor awareness of this rare disease that leads to symptomatic treatment of each manifestation. Although the first clinical sign of WS, often recognized retrospectively, is a lack of the expected pubertal growth spurt, the typical signs of WS appear progressively after puberty. Therefore, some symptoms may be absent in young patients and this can delay the diagnosis. This demonstrates that knowledge of the early signs of WS and family history can be helpful for early recognition of WS and to establish the diagnosis.

This is the first reported case of Werner's syndrome in the Colombian population, in whom clinical phenotype is similar to previously reported in other populations. We report this case to avoid misdiagnosis of this infrequent condition and allow timely identification of potential complications associated with premature aging, especially malignancies, cardiovascular and metabolic diseases.

*Learning Points*

- WS should be suspected in the presence of cardinal signs, such as voice changes, scleroderma-like skin changes, bilateral cataracts, soft tissue calcification, and appearance of premature aging.
- It is important to recognize this disease at an early stage in order to screen for and identify malignant tumors and other complications such as cardiovascular disease that are usually associated with age and can potentially threaten life.
- Multidisciplinary management of these patients is essential for the treatment and prevention of associated complications.
- Prognosis is determined by severity of complications associated with the syndrome, such as myocardial infarction, insulin resistance, and cancer risk.
- It is recommended that WS patients must be monitored with annual glucose and lipid profile, ophthalmologic examination, and complete physical examination to detect possible early manifestations of the most common complications in WS.

## Supplementary Materials

Supplementary Materials
* S1*:* WRN* gene sequencing report. Description of the methodology and interpretation of the pathogenic variant found.
